# Developing an evidence-based framework of contemporary character development at Outward Bound Schools

**DOI:** 10.1371/journal.pone.0351185

**Published:** 2026-06-12

**Authors:** Jim Sibthorp, Nick Rushford, Pete Allison, Theresa Melton, Sarah Wiley

**Affiliations:** 1 Department of Parks, Recreation, and Tourism, University of Utah, Salt Lake City, Utah, United States of America; 2 Department of Recreation, Park, and Tourism Management, The Pennsylvania State University, State College, Pennsylvania, United States of America; 3 Department of Parks, Recreation, and Tourism Management, Clemson University, Clemson, South Carolina, United States of America; 4 Outward Bound International, New York, New York, United States of America; UKM: Universiti Kebangsaan Malaysia, MALAYSIA

## Abstract

This paper presents an evidence‑based framework for character development across the global network of Outward Bound (OB) Schools. Using the Eisenhardt multiple‑case, cross‑case theory‑building method, the study examined 11 OB Schools operating in diverse cultural and operational contexts to understand how character development is fostered worldwide. Analysis revealed five key “levers” that were associated with character development opportunities: educational philosophy, service, authentic adventure, educational models, and instructor behaviors. These levers vary by context yet appear most potent when intentionally aligned to create coherent educational experiences. The resulting framework provides a practical tool for OB Schools seeking to strengthen character development through aligned design and implementation strategies. More broadly, this work advances the field of character education by highlighting both the distinctive strengths of outdoor experiential learning and the ways a global educational organization can balance overarching pedagogical coherence with the need for local adaptability.

## Introduction

Outward Bound (OB) is an international outdoor education provider with Schools in 35 countries and locations on six continents. Since 1941, OB has aimed to develop young people’s character through challenge and discovery [1]. Co-founded by Kurt Hahn, OB is grounded in Hahn’s philosophical stance that young people must be impelled into learning experiences that seek to develop their character through adventure, service to others, introspection, and compassion [1,2].

The term ‘character’ itself is complex, and its evolution at OB spans much of the 20th century [3,4]. The definition and conceptualizations of character used in this study stem from the work of the Jubilee Centre for Character and Virtue. This work conceptualizes character through a neo-Aristotelian lens, defining character as the practice of moral, civic, performance, and intellectual virtues, which, through the process of practical judgment (phronesis), are the right or just action for a given situation [5]. The Jubilee Centre’s model of character education, where character is caught, taught, and sought, outlines how educational organizations can design more intentional opportunities for character development [6] and illustrates the progress academic fields have made in advancing the science of character [7].

Yet, despite this progress, some of the character education approaches endorsed in the Jubilee Center’s model are less common in OB’s pedagogy. Formal character curriculum is rarely used in OB Schools, and attendance to topics like virtue literacy remains atypical. Instead, OB’s approach to character development is rooted in the philosophy of Kurt Hahn, and considers character development through a pedagogy that prioritizes challenges, expeditions or journeys, and elements of service to others. Given this potential divergence between the science of character and OB’s historical approach to character, we wanted to create a model, informed by the science of character, that captures how OB Schools currently foster character.

Therefore, the primary aim of this study was to better understand how OB fosters character development worldwide, given its application across diverse cultural contexts. Embedded in this aim was understanding how character, the central encompassing outcome of OB participation, may be conceptualized differently across OB Schools and how the way character is developed may differ across contexts. Using a multiple-case, cross-case study design, researchers examined OB Schools worldwide to understand the primary OB approaches to character development. Using the Eisenhardt method of cross-case theory building [8], we developed propositions most associated with opportunities for character development that integrate with OB pedagogies.

## Outward Bound’s approach to character

OB was founded on the premise of serving as a short-term character training school for merchant sailors. The original course design was a 28-day training course that focused on physical conditioning, an expedition on the school’s sailing vessel, an individual project that encouraged youth to improve something on the school grounds or for the community, and service often in the form of rescue training [3,9]. These core principles, physical challenge, expedition, craftsmanship, and an element of service, evolved as the school progressed and grew across the U.K. and Europe in the 1940s and 1950s.

By 2025, OB had established Schools in 35 different countries and had adapted delivery to different geographic, cultural, and economic environments. From the U.K. to Oman, Singapore to the United States, Brazil to Finland, OB is a global education organization. Today, Outward Bound International (OBI) provides oversight and coordination among the Schools and facilitates the efficacious delivery of OB programming. Central components of contemporary OB pedagogy are included in the OBI People, Places, Process (PPP) approach to student learning and development [10]. The 3Ps approach refers to the distinctive, interdependent elements that define an OB program. Together, the 3Ps create the conditions for maximum impact, fostering personal and social development (i.e., character) through meaningful, transformative experiences that aim to develop outcomes related to connections to self, connections to others, and connections to the natural world.

The OBI PPP approach also provides a foundation for understanding the aspects of course that contribute to student learning and character development. People refers primarily to OB staff. Places reference the natural environments where OB courses typically take place. Process describes the focus on experiential learning techniques, including the use of learning models and reflection. The approach is presented as a Venn diagram. At the intersection of People and Places lie technical skills essential to safety and risk management. At the intersection of People and Process are the interpersonal skills needed to develop a functioning group through communication, connections, and belonging. Finally, at the intersection of Process and Places is authentic adventure, or how the natural environment and experiential learning combine to create impactful experiences through challenges.

Within the PPP approach, we also considered pedagogical decisions made during the course design and implementation phases. For purposes of our analysis, design decisions occur before the arrival of the students and include the overall planning and structure of the course, such as length, which activities can be included, and the general operating area. Design elements are typically inflexible after a course begins. In contrast, implementation decisions are made after students arrive and are often based on the instructor’s judgment about how best to adjust and deliver course activities to meet students’ needs. While the extant OB literature and the OBI PPP approach provide broad insights into how OB is delivered across the global network, neither fully accounts for how character is conceptualized or targeted for development, particularly within the cultural context where the OB School is located.

## The present study

Given the existing literature on the OB pedagogical approach and conceptualizations of character, we conducted multiple case studies of OB Schools worldwide to explicate how OB Schools effectively target character through course design and implementation. We were especially attentive to the cultural and contextual diversity of the OB global network and how such diversity could be honored in our results.

## Method

This study was reviewed by The Pennsylvania State University (Penn State) IRB (STUDY00020726 and STUDY00022293). Written Consent was obtained via REDCap, administered by Penn State, for portions of the study (photo voice) involving minors. Most of the study protocols were observational or involved conversations about the program (not individual) and were determined to be Exempt by Penn State.

The Eisenhardt method of theory building through a multiple-case cross-case comparison has several distinct features that improve the robustness of findings (Eisenhardt, 1989 & 2021). First, armed with a working understanding of the character literature [6] and the OB literature [3], we developed research questions and foci. It is important to have existing literature guide, but not to determine the comparison process. Leaning too heavily on existing theory prior to data collection can skew observations during cross-case comparison and introduce researcher bias [11].

Second, we carefully selected the cases. Case selection should include variation in case characteristics to ensure that any phenomenon that emerges from cross-case data is valid across multiple distinct cases [12]. Applying this principle to the OB network meant choosing Schools that represented large deviations in the operating context, including cultural variations, programming types, funding sources, and client goals. Further, we focused on programs serving youth (ages 12–25), as this population represents the central focus of OB as a youth education program (3).

To purposively select cases, we distributed a two-phase survey to the OB global network through OBI. The survey phases were determined to be Exempt by The Pennsylvania State University Office for Research Protections. The first phase survey (n = 39; several countries have multiple Schools) sought School-level descriptive information (e.g., years in operation, size of programming, types of programming). The Phase 2 survey was a staff-level survey, with 116 Staff responses representing 23 OB Schools. Staff were asked about their experience working at their OB School so that we could better understand the nature of the students and the prioritized character strengths to inform our selection of case study Schools. Phase 1 surveys were completed between 07/12/2022 and 30/1/2023, and the Phase 2 surveys were completed between 01/03/2023 and 16/05/2023 to inform case-study selection; participants completed an informed consent document on the first page of the online survey forms.

Schools participating in Phase 2 were organized according to the Inglehart-Welzel World Cultural Map Region [13]. Combined with the World Values Survey [14], the Inglehart-Welzel World Cultural Map assisted in selecting Schools from diverse cultural regions. We used this WVS classification system to intentionally choose at least one School in each of the Eight Inglehart-Welzel World Cultural regions. This allowed the selection of cases by seeking polar types and common-process design principles [12]. While Oman does not have data in the WVS, we estimated that it would be in close proximity to Qatar and Saudi Arabia and represent the African-Islamic region.

After considering the Phase 2 survey data, we consulted with OBI on which Schools had the operational capacity to support this research effort, which would involve staff travel, hosting researchers, and involvement with this project for several years. We also considered when Schools were founded, as we sought a cross-section of older (1940s, 1950s, and 1960s; n = 4), mature (1970s, 1980s, and 1990s; n = 3), and newer (2000 + ; n = 4) Schools. These efforts created some of the boundary conditions, per the Eisenhardt method, on inclusion and exclusion criteria (e.g., official OB Schools with a capacity to support the project, culturally diverse, geographically diverse, and ranging in age, size, and funding). We chose to include 11 Schools as case studies: Brazil, Chesapeake Bay (USA), Croatia, Germany, Hong Kong, New Zealand, Oman, Romania, Singapore, OB Trust (United Kingdom), and Vietnam (see Table 1 for an overview of included Schools).

**Table 1:** Overview of Case Study Schools.

OB School Name	Year Founded	Inglehart-Welzel Region (WVS)	Total Students Per Year*	Total Students 12–25 Years Old*	Shortest Course Length (Days)	Longest Course Length (Days)	Total Program Days for Students 12–25 Years Old*	Primary Source of Revenue
**Brazil**	2000	Latin America	1149	450	2	20	1509	Students or Their Families
**Chesapeake Bay (USA)**	1986	English Speaking	6000	4800	1	12	8400	Foundations/Nonprofits
**Croatia**	2005	Catholic Europe	1500	1200	1	10	3200	Grants or Donations
**Germany**	1951	Protestant Europe	7500	6000	1	12	22400	Students or Their Families
**Hong Kong**	1970	Confucian	6800	3718	1	18	16885	Formal Education Districts or Schools
**New Zealand**	1962	English Speaking	1800	1170	5	21	20,000	Grants or Donations
**Oman**	2009	African Islamic	4000	3200	1	5	10,000	Other (Corporate Social Investment)
**Romania**	1993	Orthodox Europe	2000	1600	2.5	10	5600	Students or Their Families
**Singapore**	1967	West & South Asia	22000	20000	2	21	95000	The Government
**OB Trust (United Kingdom)**	1941	English Speaking	25000	21000	3	19	108000	Formal Education Districts or Schools
**Vietnam**	2016	West & South Asia	4000	3200	3	5	16000	Formal Education Districts or Schools

* These figures were estimated by School employees completing a survey based on knowledge of average operations.

The site visit phase, which included observations and interviews, was determined to be Exempt by The Pennsylvania State University Office for Research Protections. Consent and assent for participants at each School were collected prior to and during the case study visits, which occurred between 03/05/2023 and 20/07/2024. Written consent from parents or legal guardians of youth enrolled in the observed courses was collected via REDCap; all other consent (adults) and assent (youth) agreements were collected verbally in person by members of the study team while on location.

Third, we used multiple researchers to ensure observed phenomena were triangulated between researchers, and the interpretation and meaning-making were shared. Two researchers conducted nine of the eleven case study visits. The initial visit to OB Singapore was conducted by all four of the designated site visitors (i.e., site visit team) to allow cross-training on the research processes. A single researcher conducted the final site visit to OB Brazil due to a last-minute scheduling change. During site visits, the two researchers were often with different groups of students and staff. This decision provided a within-School comparison, which helped researchers to validate observations at the School and network levels simultaneously. This further enabled researchers to practice constant comparison of emerging observations [8].

### Efforts to mitigate bias

We used three additional methods to mitigate researcher bias. The first involved conducting interviews with knowledgeable experts who have worked in multiple OB Schools across the global network, which helped the research team reassess our Western orientation toward OB Schools (interviews were recorded and transcribed). This method provided a broader and more culturally nuanced understanding of how Schools operate and adapt to contexts across the network, challenging our preconceived notions before the case study visits. The second method used photovoice methodology [15], in which we provided the observed youth with cameras and instructed them to capture “character in action.” Toward the end of each program, we solicited thoughts from the group on particular photos taken by the youth to represent character in action on their course. This gave students opportunities to narrate course experiences, describe group processes, and articulate what was happening in their own voices. Photovoice sessions were recorded and transcribed. The third method was to employ embedded co-researchers from each case study School in the research process. These School liaisons helped the research team select appropriate courses to observe, clarified and contextualized observational data, and encouraged a culturally responsive interpretation. They also reviewed case-study notes, survey data, and our preliminary themes as a form of member-checking to enhance the trustworthiness of our conclusions.

### Analytic process

After completing site visits, we developed a case study file which referenced field notes, operational context, and relevant images, artifacts, recordings, or transcripts. These files were created by the site visit team and were reviewed by the liaisons from each School for accuracy, clarifications, and input on any observations that did not fully reflect the School’s perspective. This process helped the research team to refine our thinking and better understand the distinct operational contexts of 11 case study Schools.

After collating the data and refining our perspectives with liaison input, we conducted reflexive thematic analysis within and across cases [16,17]. First, each individual on the research team reviewed case study reports individually, becoming familiar with the data present within and across all case studies. During this review, each individual constructed themes based on their interpretations of the data. Next, the research team came together in person to discuss the codes that were generated from individual researchers, discussing similarities and differences across the team.

From this exercise, we proceeded to axial coding, where codes were grouped into initial themes based on relationships across codes. For example, codes related to “mealtimes,” “rapport” between instructors and participants, and the role of “reflection” were found to relate to an overall theme related to instructor behaviors, whereas codes related to specific tools used by instructors, such as Frame, Frontload, Action, Review, Transfer (FFART) and Training/Main/Final (TMF) were connected to a separate theme related to models. Likewise, codes related to “intentional programming,” “purpose,” and “ethos” connected to a theme related to educational philosophy. Those themes were then checked against the data that came from the case studies and were refined through discussion until consensus was reached across the research team. These consensus themes were presented to each of the 11 liaisons at a two-day in-person meeting to discuss how the themes related to one another to inform propositions as a member checking process. Following this member check, and consistent with the Eisenhardt Methods, this process concluded with a framework centering on five propositions that helped describe the ways in which Outward Bound Schools proactively design and implement their programs across varying contexts to afford prioritized character development opportunities.

### Positionality and reflexivity

While making efforts to be objective and mitigate bias, we acknowledge that the research team’s positionality shaped data collection and interpretation. The research team brought extensive professional and scholarly experience in outdoor and experiential education, with several authors having longstanding affiliations with OB programming. We represent an interdisciplinary team with terminal degrees in education, human performance, and developmental science. This positioning provided important contextual knowledge that informed study design, access to sites, and interpretation of pedagogical practices. In many cases, it allowed us to gain an emic perspective on how the Schools operated. At the same time, it introduced the potential for assumptions shaped by prior exposure to OB philosophies and Western traditions of outdoor education. We were particularly attentive to how our positionality as primarily Western-trained researchers, affiliated with academic institutions and, in some cases, the OB network, might influence both data collection and interpretation. For example, initial understandings of character development and effective pedagogy were informed by existing literature and prior experience, which risked privileging certain conceptualizations of character. To mitigate this, we intentionally selected case study sites across diverse cultural contexts and engaged in ongoing reflection about how local meanings of character and educational practice might differ from our own expectations.

Finally, we acknowledge that the interpretation of the data, particularly in cross-case comparison, was influenced by our analytic goals of theory building using the Eisenhardt method. While this approach required abstraction across cases, we remained attentive to preserving contextual nuance and avoided privileging any single value system. Through these combined strategies, we sought to balance the benefits of our insider knowledge with critical reflexivity, enhancing the trustworthiness and credibility of the findings.

## Situating the results

Through the case studies, we observed that all OB Schools aim to develop character, but they may target different character strengths in response to their context (defined below). Character development research has identified the importance of explicitly targeting and prioritizing character outcomes through programming [6,18,19]. Given the history of OB, the measured outcomes included in their program evaluation efforts (e.g., resilience, self-confidence, social-competence, compassion, and environmental responsibility), photo-voice responses, and the outcome focus we generally observed, “character development” (definitions and foci varied) was prioritized at all OB Schools observed. Further, Schools typically had a shared language regarding prioritized outcomes and clear behavioral expectations for students. Before explicating the levers OB Schools selectively employed to instill character, it is important to acknowledge design and implementation elements generally considered necessary to character development and universally present.

First, relationships are central to character education [6,19]. A focus on relationship development that intentionally allows for strong social bonds to form among the students and between the students and the instructors/trainers is a hallmark of OB programming and was universally observed. The instructors valued rapport with their students, and the design and implementation of the OB program generally facilitate relationship skills and social development. This approach to relationships generally created a safe climate within the course, prioritizing valuing others and creating a climate of support and respect.

Second, experiential learning is essential to habituating character strengths [6,19]. OB courses, by design, are experiential and practice-based. Students were asked to build skills to overcome challenges through a course arc that has a clear beginning, middle, and end. Through this arc, they are impelled to practice aspects of character as prioritized by the School using cooperative, reflective, and experiential learning.

While the above approaches to character development were ubiquitous across the OB network, other approaches were more selectively employed by Schools. We have chosen to label these approaches “levers.” We chose this language as the contextual boundary conditions, which are further explained below, varied by School. Consequently, Schools must decide how to maximize the student experiences, considering that some aspects of the course experience are flexible while others are not. We began using the analogy of a musical mixing board, where the sound engineer adjusts various levers to produce a coherent and pleasing musical piece. Similarly, OB course designers aiming to develop character might choose to emphasize certain adjustable course elements, or levers, to best meet the needs of their students and clients. Just as a sound engineer tailors music based on inputs like musicians and instruments to suit an audience, OB course designers use these levers to appeal to their audiences. These levers are employed differently across the network to ensure each OB School remains viable and valuable within its current operating context. Thus, while all Schools are creating music, they do not all create the same music or use the same instruments. When levers are wisely adjusted, they create coherence in the programming, which was associated with opportunities for character development in our observations at OB.

## Evidence for a character framework

Consistent with Eisenhardt’s theory-building approach, our goal was to identify recurring patterns and propose relationships among constructs, rather than to test causal effects or evaluate the relative effectiveness of specific practices. Thus, in the following subsections, we explain each of the five levers, worded as testable propositions. These levers include having an *educational philosophy*, including *service* and *authentic adventure* in course designs, and implementing courses using *educational models* and *instructor behaviors*. These levers are most effective when intentionally aligned to create a coherent product. Each subsection provides examples from our data. Before discussing each lever, it is necessary to understand context as a key boundary condition shaping how Schools operate.

### School context: The key boundary condition

We found the complexities of context, including cultural differences, operational constraints, funding structures, and client bases, to shape how Schools conceptualize character, making operating context the key boundary condition. Given these different conceptualizations and concomitant priorities, Schools work within their structural or fixed constraints by adjusting the most salient and malleable design and implementation levers. A School’s operating context shapes how the School’s key actors negotiate its educational philosophy, which, in turn, determines which character strengths it prioritizes. Table 2 provides an overview of the case study sites and courses observed to better compare contexts.

**Table 2:** Case Study Site and Observation Overview.

OB School Name	Population Type	Population Overview (number of students, age, gender)	Days of Course	Funding of Course	Courses Format & Activities	Instructing Format
Brazil	Social Program and Open Enrollment	14 students; 15–18 y.o.; mixed gender	10	NGO and Families	Expedition – Backpacking	2 instructors per group, always with group
Chesapeake Bay (USA)	Public charter school	Group A: 6 students, 13–14 y.o.; all boys, 1 teacher Group B: 7 students, 13–14 y.o.; mixed gender, 2 teachers	5	Formal education	Expedition – Backpacking, rock climbing, solo	2 instructors per group, always with group
Croatia	Social Program and Open Enrollment	11 students; 14–18 y.o.; mixed gender	7	NGO and Families	Expedition – Backpacking, rock climbing, ropes course, service, solo	2 instructors per group, always with group
Germany	Private high school	Group A: 11 students, 16 y.o.; mixed gender, 1 teacher Group B; 10 students, 16 y.o.; mixed gender, 1 teacher	6	Formal education	Center-based with one night hut expedition- Initiatives, hiking, solo, climbing,	1 instructor, generally with group 9am-6 pm
Hong Kong	Public school	Group A: 11 students, 15–18 y.o. Group B: 11 students, 15–18 y.o.	5	Formal education	Expedition – hiking and kayaking, solo	1 instructor per group, always with group + activity specialists
New Zealand	Open Enrollment	Group A: 10 students, 18–25 y.o.; mixed gender Group B: 10 students, 18–25 y.o.; mixed gender	21	NGO and Families	Center-based and expeditions (schemes) – backpacking, sailing, rock climbing, solo,	2 instructors per group, live at center, always with group
Oman	Education to Employment and Public School	Group A: 14 students, 24–30 y.o.; all women Group B: 20 students, 15–17 y.o.; all boys, 1 teacher	4	Corporate Social Investment	Expedition – initiatives, camping, hiking	2 instructors per group
Romania	ERASMUS course and School	Group A: 28 students, 15–16 y.o.; mixed gender Group B: 17 students, 11–13 y.o.; mixed gender, 3 teachers	A:10 B: 3	NGO-nonprofit and Families	Center-based and expeditions – hiking, camping, rock climbing, initiatives,	1-2 instructors per group, live at center
Singapore	Ministry of Education program	Group A: 14 students, 14–15 y.o; mixed gender Group B: 14 students, 14–15 y.o.; mixed gender	5	Government	Center-based – orienteering, dragon boating, camping, ropes course, kayaking	1 instructor, always with group
OB Trust (United Kingdom)	Independent and Public School	Group A: 12 students, 13–14 y.o.; mixed gender; 2 teachers Group B 12 students, 12–15 y.o.; mixed gender; 1 teacher	A: 7 B: 5	Formal education	Center-based with one night hut expedition – initiatives, ropes course, gorge walk, hiking	1 instructor, generally with group 9am-9 pm + high adventure specialist
Vietnam	International School	10 students, 14–15 y.o.; mixed gender; 1 teacher	5	Families	Center-based and expedition – hiking,	2 instructors per group

The target client base influences which aspects of character are prioritized in a School’s programming based on desired outcomes. Different client groups and types of programs inform the Schools’ structures and philosophy. For example, clients from educational districts or formal schooling often seek outcomes that align with academic achievement, focusing on performance character strengths like resilience and grit, or intellectual character strengths like curiosity and creativity. Other clients, such as governments or community foundations, may prioritize building cooperation or compassion, which are considered civic or moral character [6].

The location of a School significantly influences programming design decisions made by administrators. Key characteristics of the location include geography and proximity to urban areas. Schools with access to immersive natural environments can easily incorporate authentic adventure activities. For example, the New Zealand basecamp facilitates day and overnight trips directly from the property. In contrast, Germany has fewer areas where tent camping is legal. This limitation, combined with the nearby Alps, encourages using alpine huts, which are culturally appropriate and common in Germany.

Conversely, Schools located in urban areas may rely more on educational models and expert instructional behaviors to afford character-rich outdoor experiences without high adventure. For instance, due to insufficient access to wilderness, OB Singapore designed a program that uses urban greenways and camps in community parks to serve the nation’s youth.

How courses are funded, like client base, can determine priorities within programming. Funding is often influenced by a confluence of the School’s cultural fit within the national context and its governance structure. Schools like Oman and Singapore, with strong industry or government support, collaborate on outcomes that are beneficial to local society, workforce, and citizenship development. Predictable program funding allows for stability but can limit diversification. When a School is funded through a wider combination of grants, donations, and/or students’ families directly, such as in Schools like Croatia and Romania, funding is less consistent, and interests are more varied. While the downside of inconsistent funding is self-evident, the benefit is that the consumer or donor is bought into programming vision and outcomes, and the programs remain nimble to adapt to client needs.

### Levers of character development: Key propositions for design and implementation

Through our observations, we propose five overarching levers that vary in both breadth and depth of use across the network. Each of these is stated as a testable proposition, with the goal of informing further research efforts on how character can most effectively be developed through OB. These propositions reflect patterns observed across cases, including variation in how levers were emphasized or enacted, rather than prescriptive or evaluative claims about best practice. These levers include *educational philosophy, service, authentic adventure, educational models,* and *instructor behaviors*, which includes three sub-categories. Each lever is discussed in additional detail below.

#### Educational philosophy proposition.

*Having an educational philosophy that is singular and explicit may contribute to character development opportunities through a coherent and consistent approach.*

We define an educational philosophy as a guiding set of beliefs and values that inform practice. Having a well-defined educational philosophy was observed as valuable for OB Schools as it provided a guiding set of principles that shaped every aspect of the organization’s learning and development approach and helped create a shared culture at the School (among all employees, regardless of their role). We observed different philosophies in different Schools, and we noted that some were more focused on different aspects of character (i.e., moral, civic, performance, and intellectual). During the research, across cases, we observed that Schools that had a singular, consistent philosophy, which was shared, understood, and implemented consistently, appeared to have a greater positive impact on students. The philosophy at these Schools seemed to provide the ‘north star’ for practices and policies in the institution and the plethora of different decisions made throughout the School.

In smaller Schools, it was often the case that educational philosophies were developed democratically and evolved over time but were consistent among all staff. In larger Schools, it is harder to involve everyone in the development of an educational philosophy. In these cases, a smaller leadership group tended to develop a philosophy and then educate the staff teams about the philosophy. In some Schools, the educational philosophy was explicitly and clearly based on Hahnian principles; in others, these principles were less pronounced. In some Schools, the educational philosophy is strongly connected to the broader political policy context; in others, it is not.

The philosophy of a School like Oman is clear and unified. In our observation, Oman’s priorities were clear. Discussions on Oman’s Vision 2040 were woven throughout courses. Staff helped students identify economic shifts in Oman and employability, with the aim of getting students to shift from government dependence for career options to more personal responsibility. A hard work ethic, personal responsibility, and resilience were prioritized outcomes.

Schools with fewer observed opportunities for character-related learning often had an unclear educational philosophy or philosophies, or staff had been given too much latitude to develop their own philosophies. Some Schools borrowed approaches from other Schools that were misaligned with their School’s philosophy and were ineffective. Several of the newer Schools we visited wrestled with copying curricular and training materials from more established Schools, lacking the philosophical through-line to adjust and adapt practices to their distinct operational context. This lack of coherence may create confusion for students who experience inconsistent implicit and/or explicit messages. There were also inconsistencies within the School itself in terms of messaging and focus. One School, for example, promoted outcomes externally that were not aligned with the priorities embedded in staff training, suggesting a gap between the School’s espoused educational philosophy and its operational delivery.

#### Service proposition.

*When courses include meaningful service to others or the environment, character development opportunities appear to be more salient within the observed cases.*

We define service as an intentional act of contributing time, effort, or resources to benefit others or the world around us. Service observed included service to nature, the OB group/center, or the broader community. OB courses, historically, have included a service component [1,3,4]. However, observations made during the study suggested variability in the role and form that service had on courses. Several Schools have strategically excluded service in their programming, whereas others have incorporated it in small ways (e.g., picking up trash during expeditions observed in Germany and UK cases). While serving others outside the group can be a powerful experience for students and has historic ties to service at OB, service activities do not require coordination with external groups. For example, students can experience service when serving the group (e.g., serving meals, cleaning, and helping each other build shelters). However, service must be meaningful to students to be experienced by participants as relevant within the course context.

Meaningful service depends heavily on the context of a course, but appeared most effective when integrated into the course experience. Observations and interviews with staff highlight how non-integrated or stand-alone service can feel inauthentic and disconnected from the rest of the course experience. Often, this type of service was described as a “check box”. However, integrated service is structured into the course from the beginning and in such a way that the acts of service are framed and embedded in course functions. Examples of this type of service include daily roles and chores, cooking meals for others, and practicing low-impact camping and travel techniques. This type of service to the group, when practiced daily and highlighted by instructors, was frequently associated with the expression of responsibility and compassion for others and the environment. When this becomes habitual, less proximal acts of service, such as picking up trash found on the trail or helping orient a lost hiker, appeared less inauthentic and more aligned with the habituated ethos of service on one’s course. An example of service becoming part of the course ethos was observed in Brazil. The whole course was framed around ‘to serve, to strive and not to yield’ (a common OB motto adapted from Tennyson’s Ulysses). This was the focus for many discussions and for accountability regarding behavior. This focus made sense given that the majority of students were from Favelas and were not short of perseverance, grit, or resilience. Thus, a focus on community and what kind of community they want to live in made sense. This emphasis led to a wide-ranging discussion, including their own group dynamics, and to increased discussion and interest in contributing to communities beyond the OB experience, such as family, faith congregation, or Favela.

Schools that did not include service as part of the course or included service superficially appeared less effective in providing opportunities for students to care for others and the environment. Schools that minimally included service often utilized simple, convenient activities (e.g., picking up trash along a trail), which often necessitated careful framing and debriefing to create engagement and meaning. While in some cases this less central approach was merely a lost opportunity, in other cases the “service” appeared tokenistic and off-putting to the students. We also noticed that programs in which the instructors did not frame or introduce the concept of service to others, whether formally or informally, often had less cohesive group dynamics, reducing opportunities for meaningful contributions to others.

#### Authentic adventure proposition.

*As courses increasingly incorporate authentic adventure characteristics, they appear to offer more frequent and varied opportunities for character-related learning within the observed cases.*

An explicit part of OBI’s PPP approach, we define authentic adventure as the immersive, challenging, and meaningful experiences (not activities) that push individuals to work near the edge of their capabilities (not comforts). Authentic adventure is rooted in real-world environments with natural consequences for the group, including instructional staff. It emphasizes physical and emotional engagement with nature and toward a goal, fostering personal growth, resilience, and character through direct, hands-on challenges. Regardless of momentary discomforts and struggles during the experience, authentic adventure is recalled as memorable and rewarding. Observations from this study identified that available elements of authentic adventure seemed to be associated with character development opportunities.

Immersive experiences involve being away from familiar contexts, removed from existing social structures, and limited in one’s distractions. The novelty of the immersive experience was highlighted in an observation from Hong Kong. On this course, most of the students had very little, if any, outdoor experience and were not used to so much time outside, camping, trekking or sea kayaking. Thus, teaching had to cover everything from applying sunscreen to bag packing, walking, hydration, and tent pitching. Not only was the natural environment novel for most young people, but also the hiking, group work, and cooking meals together. This was all carefully choreographed so that the experience was not overwhelming but rather progressive and maintained interest and engagement.

Challenging experiences are essential to pushing students toward and near the edge of their capabilities. Once near their perceived edge, students are confronted with the reality of their ability to do more than they thought possible. By design, this often takes the form of physical challenges; however, social, emotional, mental, and even moral challenges are all possible in the course experience. Sometimes these challenges are inherent in the course design, such as within the expedition. Even on the short course observed in Oman (4 days), the expedition allowed for authentic roles and responsibilities around camp (e.g., cooking, tent set up, and cleaning). Students were challenged by carrying a heavy pack on a relatively rugged hike while navigating off-trail terrain that necessitated group cooperation. In center-based courses, these challenges can be designed into the course. For example, in New Zealand, while at the OB center, all watches start the day with a group warm-up followed by a three-mile run. These activities are completed in silence in order to avoid disturbing local residents. This happens at 6 AM and is followed by a swim in the sea. A clock is visible so students can see their improvement in time as they progress through the course, and this process builds to the course marathon on the final day.

Natural consequences are a key function of the combination of an immersed natural environment and the use of challenge in this environment. Natural consequences can take the form of inclement weather, mistakes in navigation causing route-finding errors, and the inability to light a fire with damp wood, resulting in a cold (or non-existent) dinner. Unlike imposed consequences, such as when an instructor blindfolds an outspoken student to silence their voice, natural consequences are inherent in the activities. They generally impact the entire group, including the staff, and are, at times, preventable with increasing skills and responsibilities throughout the course. For example, observations from Vietnam highlight how two heavy days of rain impacted the group. The staff embraced the challenge, but when students were struggling too much with the adverse weather and challenging terrain, the group took an extended break, and students had the chance to learn how to skip rocks. The staff spoke about how important it was that youth struggled but didn’t suffer, and that students should feel challenged yet recall the adventure fondly rather than with bitterness.

A common goal is also a key element of authentic adventure. Whether this goal is physical, such as climbing to the top of a mountain summit, or emotional/mental, such as equitably distributing group gear and food and packing up camp, shared goals create authenticity within the adventure experience. Finally, adventurous experiences are recalled as memorable and rewarding. Given the hardships commonly faced during an authentic adventure, these experiences working together toward a shared goal can be powerful levers for character development.

Schools that minimally incorporate authentic adventure appeared less effective at engaging students in real-world problems and challenges and were forced to rely more on didactic instruction rather than experiential learning to develop character strengths. Some observed “adventure” activities functioned more as recreational diversions than as part of a core educational process. In other instances, programming prioritized completing activities (e.g., finishing a hike or ropes course element) over the learning they were intended to produce. As a result, there was limited use of real situations and their natural consequences, the foundation of authentic adventure, as drivers of meaningful development.

#### Educational models proposition.

*The intentional application of appropriate educational models was consistently observed alongside opportunities for character development.*

Central to process within the OBI PPP approach, educational models serve as guides for designing teaching and learning in courses. We define educational models as structured frameworks or tools that guide the design and implementation of teaching and learning experiences and promote student engagement, progression, and offer a common language for instructors and students. Commonly utilized models seen during observations include Frame, Frontload, Action, Review, Transfer (FFART), a Comfort Zones model, Training/Main/Final (TMF), and an Experiential Learning Cycle. These approaches offer structured pathways for students to engage in progressive and often transformative learning experiences.

We observed educational models utilized at several levels or planning phases of courses, which allows for building appropriate progressions. At the broadest level, models can shape the overall delivery of courses starting in the planning phase. This manifests through the course design of activities and itineraries to scaffold opportunities for character development. For example, TMF can be used to design a course’s itinerary to provide increasing opportunities for student responsibility, autonomy, and challenge. Models are also used in the day-to-day planning and implementation of courses. Each day can be structured using an educational model. For example, FFART was used by instructors to sequence the whole day’s activities, framing and frontloading to students the whole day’s plan, proceeding with a series of activities, and then reviewing the entire day in an evening meeting debrief. Finally, models can be used discretely for individual activity experiences. For example, a Comfort Zone model is often used to introduce and prepare students for a high ropes course experience. This model introduces concepts of edgework and our ability to grow as we near the edge of our capabilities, which are different for each student, but is the goal of the challenging activity. The scalability in the use of educational models is an important takeaway for practitioners.

The use of educational models also contributes to experiences being made meaningful, applicable, and transferable to students’ learning. This was particularly evident in the U.K. case study. Instructors at this School built courses around student strengths and pre-established “frames” that were used interchangeably depending on desired course outcomes and client base. The schoolteachers accompanying this group noted how effective the frames were and how the students picked up and used the language of the frame throughout the course (5Ps, passenger, protester, prisoner, participant, pilot; and comfort zone specifically). Another example of the effective use of models was observed in Vietnam. Students were provided a journal that they used to take notes and to align with the curriculum that the course director created. Through this journal, students were introduced to several models and common language used in their course, such as experiential learning (with the explicit goal of transferring what they learn at OB to their daily lives), the importance of stepping out of their comfort zone to facilitate learning, identifying values to focus on throughout the week, and reflecting on their experiences to understand how these experiences can support their lives outside of the program.

Less effective model use can seem disjointed, and was observed when instructors included models effective in some settings, but misaligned with their School’s course design or philosophy. This was also the case when instructors utilized multiple models that did not connect well with one another or when models seemed to be lifted and dropped from one context to another with only a shallow grasp of the models. In some cases, too many models were introduced, which diluted their utility.

As a counterpoint to the advantage of educational models, in one of the courses observed, the instructors approached the course more organically, without explicitly applying models common on other OB courses. These instructors appeared highly successful in offering character opportunities without the explicit inclusion of educational models. However, it should be noted that this course was also longer and had older students. In another case, a Comfort Zone model was combined with a “challenge-by-choice” approach in a way that allowed participants to disengage. Instructors did not feel as if they could push their students to engage in activities because students said they weren’t “comfortable” when really the students appeared more disinterested and disengaged. Thus educational models, like other approaches, need to be employed intentionally and coherently to be effective.

#### Instructor behavior propositions.

We define instructor behaviors as the ways that instructors implement courses to create relationships, build course culture, leverage instructional strategies, and adapt to student needs. Given the diversity and complexity of productive instructor behaviors, this proposition is further considered as three subparts: relationship and culture building, instructional strategies, and adaptability. We have chosen to present our propositions at the sub-category level and have detailed individual instructor behaviors in the following paragraphs.

*Instructor behaviors that prioritize course culture appear to support character development opportunities.*

Course culture and rapport with students are foundational to creating student buy-in for the experience, understanding students’ expectations and motivations, and building trust to later be used to encourage students in challenging activities. Observations from Chesapeake Bay (USA) highlight rapport building and describe the benefits of having two instructor team members for this task. Having two instructors allowed a lot more flexibility in the ebb and flow of relationships. Acting as a pair, they could switch roles from driver or disciplinarian to mentor/friend and allow the other instructor to fill in the gaps. The motto was “Firm, Fair, Fun.” Given the nature of a 5-day program, they made choices appropriately to balance rapport, expectations, and program needs. Building rapport with the students was one of the main themes of the program/goals of the instructors. The instructor team was very much part of the group, not outside the group, yet at the same time, the emphasis was on moving from “instructor-led” in the first couple of days to more student-led at the end of the expedition. Efforts were put into engaging with the students – puzzles, riddles, and games while hiking and in the evening through sharing personal stories, observations, or anecdotes.

Mealtimes represent a differentially utilized opportunity to connect with students, build rapport, and establish the group culture. Our study observations noted the role shared meals with the student group had on relationships and the course’s culture. Mealtimes at many OB Schools present downtime where course-related goals are paused, giving staff an opportunity to get to know students better without the pressures of facilitating activities or prescribed learning outcomes. An example of a robust mealtime routine was observed in Brazil. For the first few nights, the instructors took the lead on cooking and organizing meals with increasing involvement and tasks from the group as they became faster at pitching tents and setting up camp. When meals were ready, the group gathered and waited for everyone to be served. There was a pause and an appreciation of the cooks, and the meal was shared as a community with lively, informal conversations, occasionally encouraged by the instructors via a timely question or observation.

Traditions can also build culture on a course, aiding in students’ developing a sense of belonging and group cohesion. The run-and-dip is a common OB tradition, where students run and then go for a swim. Variations of this tradition were observed at several schools, including the OB Trust, OB New Zealand, and OB Hong Kong. Pinning and graduation ceremonies are likewise parts of the OB tradition that many Schools retain to create shared experiences across the larger OB community. Regionally, traditions can also be used to integrate with and capitalize on local cultures. OB Croatia does all their cooking over open fires; while this is a tradition for the School, it is also a traditional way to life in the local community. Similarly, OB Oman serves meals in a group from a single pot; students do not use utensils and pray several times each day. This integration of regional religious norms and traditions strengthens the cultural connections, both within the course and the larger community.

*Instructional strategies that are intentionally employed appear to support character development opportunities.*

Instructional strategies were common across the cases. However, we noted that several effective strategies were used differently across the observed Schools. Specifically, individual and group reflection, framing activities for context, and incorporating designated roles with concomitant responsibilities into the course.

Whether a few minutes or a few days, individual student reflection is a powerful part of the learning process and helps connect course experiences and learnings to their own way of knowing and thinking. This process can help solidify learnings and transfer learnings into post-course contexts. Observations at Chesapeake Bay (USA) documented a large variety and emphasis on individual reflection methods. Reflection was incorporated into the course design, and its structure provides ample opportunities for authentic feedback and reflection. As is common across programs, different reflection strategies work better with some students than others. Some observed strategies included personal journaling, letters to future self, and solos. The solo as an individual reflection activity was observed across many Schools, with some Schools using shorter solo moments (30–60 minutes) as a pause between activities and other Schools facilitating overnight solos.

Similar to individual reflection, group reflections help student groups process and solidify their learning. However, the function of the group in this shared process contributes to learning about interacting with others, teamwork, and individual roles within group work. In the short term, group reflections can aid groups in increased performance on subsequent activities. Long-term, these reflections can help students understand how they work in groups and increase their ability to communicate, collaborate, and connect with others. An example from the Romania observation describes how students are offered the opportunity to give constructive and kind feedback to one another, and they are given non-verbal opportunities to reflect on one another. In one observed activity, students were instructed to “touch the shoulder of the person who helped you the most today.” Another observed activity included a “fish tank,” where members of one team provide feedback to each other in front of the larger group, then the larger group is able to add to the feedback. This is done to help students get a more honest, authentic picture of themselves.

Instructors who can frame a need for each activity are more effective at progressing students through meaningful learning in the course. Almost any activity can be framed in multiple ways. Knowing how to set up and frame an activity so it positively and meaningfully contributes to the students’ experience and learning is an important instructor behavior. Hong Kong’s course was framed through an intentional tone set. When individuals introduced themselves at the beginning of the course, they were also prompted to share something about their character and what they wanted to change about themselves over the week. For example, one student shared that they could have a temper and be lazy and wanted to change that this week. The goal was for students to push each other. They then closed the framing by jumping off the jetty into the ocean to signify the beginning of the transformation.

Through the use of roles and chores, opportunities for service within the group aid in developing responsibility and compassion for the group members. Roles were common across case studies, but how they were used varied in centrality, responsibilities, and number. One common role was student leader of the day. Chesapeake Bay (USA) in-field resources demonstrate clear roles and responsibilities, including: Leader of the Day (navigation and time management), Chefs (cooking), Survivalists (bear hang, hydration, camp sweep), and Scrubbies (organize kitchen, clean dishes).

*Courses that are adaptable to students’ needs appear to support character development opportunities.*

Even the best-laid plans might fall apart when the student group arrives, with their expectations, motivations, goals, and strengths. Instructors are, therefore, instrumental in their ability to adjust the plan and the whole course design to the needs of the student group in front of them. For example, if a group is getting along well and exhibiting a high level of connection and cohesion, an Instructor might opt to skip some icebreaker initiatives and move into more challenging problem-solving tasks early in the course. On the other hand, a group that is struggling to pack up and leave camp on time might benefit from a rest day and some individual coaching on backpack packing and how to help one another with shared tasks. Observations from Croatia detail the benefits of instructor autonomy during an expedition. When students were supposed to cross a river, instructors realized that the river was much higher than anticipated, given the recent rain. Therefore, the challenge was much more difficult than anticipated and involved bush-pushing (off-trail travel) and additional navigation, as students had to find another way over the river. This was a big challenge for several students. One instructor offered, “That is not what we planned, but it is what happened,” which reflects the flexible nature of the course.

Operating constraints can reduce flexibility in time management. Time management is an effective lever when instructors can adapt it to the needs of the student group. Courses with a high level of authentic adventure, which might be journey-based and remote, might find that the timing of the day must accommodate not only the day’s objectives, but also weather, level of group cohesion, and skill, among other factors. If the route is easy, the pace of the day can be quite relaxed, perhaps enabling more focus on group dynamics and connections or formal lessons. If the route is challenging or the weather is adding too many hazards, the management of the day may be stricter and more instructor-led. Conversely, courses with lower authentic adventure may be subject to stricter time schedules to accommodate a sample of activities, including firmly set mealtimes at a dining hall in a center-based program. In such settings, time management must be stricter to adhere to the schedule. This also creates opportunities for responsibility within the student group for timekeeping and timeliness. A balance of strict and flexible time management was observed at Chesapeake Bay (USA). The five-day schedule and some immovable programming (e.g., the climbing day) made time management central to the program. However, staff had great flexibility within windows (e.g., on an expedition day). They could delay lunch until as late as 2:00 to accommodate AM activities, and could eat dinner late if the day’s activities dictated when they got into/set up camp. The staff used a group journal to ensure most courses included the intended elements. This provided structure to the student leaders of the day without constant instructor direction. High-functioning groups could use this journal to manage their flow through the day with less instructional support as the course progressed.

Like other instructor behaviors, facilitation styles are most effective when they are tailored to student needs. This might mean leveraging certain leadership styles or voices (directive or democratic) to encourage student involvement or activity success, depending on the activity learning outcomes. The facilitation skill of OB staff was captured during observations in Germany. Below were researcher notes throughout the observed course, which describe the variety of techniques and attunement of instructors to the needs of the group.

- During this activity, the instructor provided the students with a walkie-talkie to further give the impression that students were leading alone (although they remained in eyesight, and he became more involved when the hike turned more technical).
- The instructor stayed a bit removed from the group, friendly but not central, in an effort to force the group to work together and support one another.
- Instructors take care to manage the group dynamics; given the shorter nature of the course, they are careful not to push the group dynamics to a point where they cannot find resolution prior to the end. This requires observation and for the instructor to alternate between giving the group space to work through things and getting more involved when tension feels high.
- The staff is incredibly skilled at “attention bending,” ensuring that the discussion relates to the lessons and needs of the group. They are highly skilled at choosing appropriate activities for the group and using them based on group needs.

Similar to educational models, instructor behaviors that were mismatched with other levers at play were observed to offer fewer opportunities for students to consider character development. Often, the other levers at play, such as authentic adventure or service, which are commonly designed into a course, seemed harder to adjust, while adjusting specific instructor behaviors might be more flexible. Instructors who tended to statically utilize certain behaviors, regardless of the course type, student group, or course outcomes, were less effective. An example of this mismatch was a course structure with a high need for facilitating activities, with little downtime for getting to know students, and then instructors opting to have mealtimes separate from students. Sharing mealtimes in this scenario would have been a good opportunity for rapport building outside of highly structured activities. Another example was when instructors would minimally frame activities, that were important to frame, to connect them to larger educational models in use. When using the FFART model, failing to frame an activity, or debrief and connect to a subsequent activity, lessens the effectiveness of the activity itself.

## Synthesis and implications

**Fig 1:**
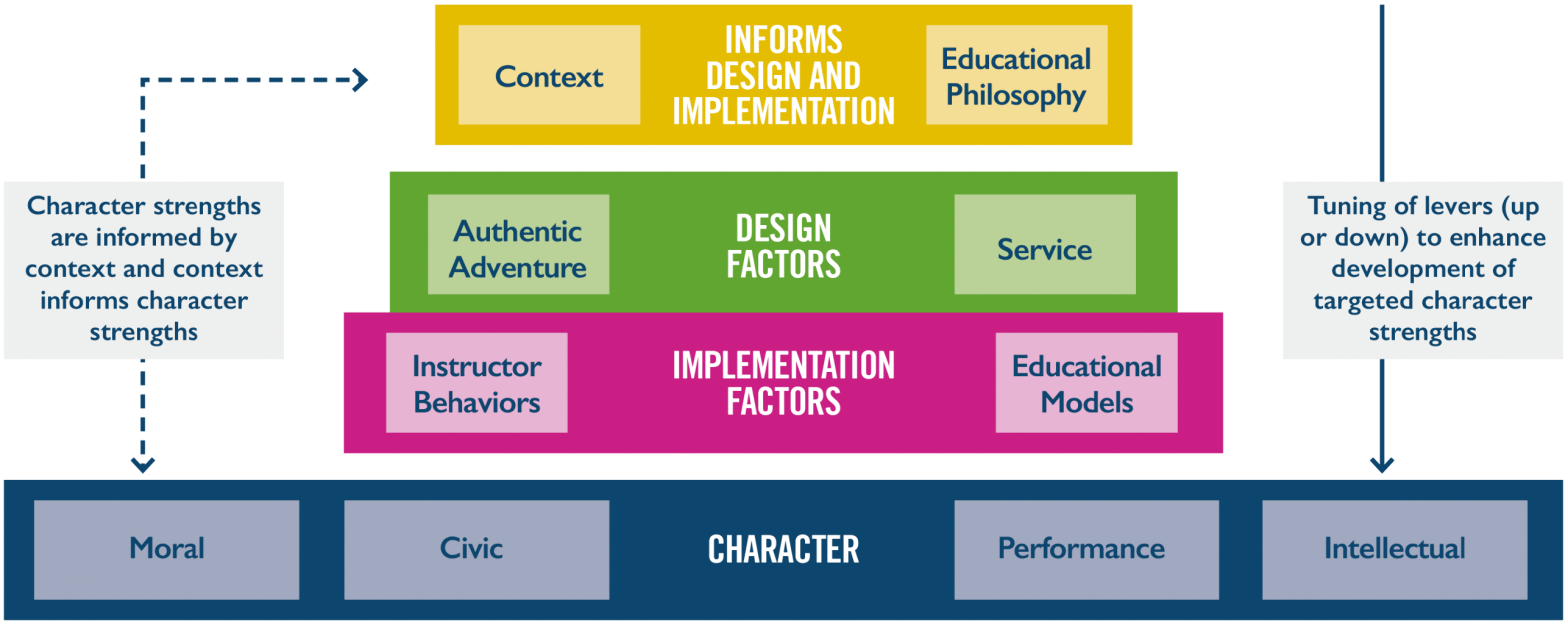
Theoretical Framework for Promoting Character Development at Outward Bound.

We have detailed context as a key boundary condition and offer five overarching propositions that emerged from cross-case comparisons that theorize how OB Schools can leverage character development opportunities in programming. These levers include 1) defining and designing programming through a unified educational philosophy, 2) incorporating meaningful opportunities for service, 3) increasing authentic adventure characteristics appropriately within context, 4) integrating helpful educational models, and 5) encouraging beneficial instructor behaviors to match student needs. Fig 1 below presents the theoretical framework linking these five propositions, which helps to understand the relationships between these levers and character development.

We found that different conceptualizations of character existed, but those differences were not just related to the culture of the country. Instead, many other factors impacted character conceptualization, particularly those detailed in our context boundary condition. There were even specific instances where the School’s culture was actually counter-cultural, filling a niche unavailable within the country’s formal education system and more aligned with OB as an organization than the country in which the School operated. This finding is in line with other work, which explains the interplay between national and organizational culture [20].

Given these results, the empirically derived theoretical framework starts and ends with character strengths, as Schools may target specific character strengths through their programs. For example, government-aligned Schools like Singapore are well-positioned to develop civic character and help youth understand their role in contributing to the nation. However, our findings suggest that the influence is bidirectional, as character strengths prized in specific countries and cultures shape the context within which a School programs, and this context informs the educational philosophy.

For example, in Germany, the School acknowledged the tension between pushing students to the edge of their capabilities and ensuring students have fun and enjoy their time at OB. In a country with many options for outdoor education, customer satisfaction is an important factor in this School’s financial context. Therefore, Germany’s philosophy must account for this tension. Pushing students to experience discomfort at the expense of enjoyment could jeopardize the School’s enrollment; however, if the School goes too far toward accommodating customer demands, it risks undermining its ability to deliver impactful programming. A School’s philosophy is shaped in the operating context. Together, the context and educational philosophy levers are adjusted to respond to targeted aspects of character, which informs how a School designs and implements courses.

The design of programs is where the authentic adventure and service levers are adjusted to align with the context and philosophy. The degree of authentic adventure is informed by contextual factors such as School location. Access to programming locations, camping, trekking, and waterways are all factors that inform the way a School might best leverage authentic adventure. Design for high levels of authentic adventure would be well aligned at a School that values physically challenging journey-based expeditions, and also has easy access to wilderness. In similar ways, service is informed by context. Integration with local communities yields stronger collaborations, which increase the meaning of service partnerships and projects. Design for high levels of service would be well aligned at Schools with close neighbors, that value community engagement, and which coordinate projects for each course that strengthen these connections. Authentic adventure and service are key levers that require design before a program begins. As context and philosophy inform these two levers, authentic adventure and service design subsequently inform instructor behaviors and educational models through how courses are implemented.

The implementation of courses relies on the appropriate use of prioritized and maleable instructor behaviors and educational models. The tuning of these levers is course and student need specific. While certain instructor behaviors and educational models generally work well within certain philosophies or authentic adventure levels, instructors’ judgment on the course is needed to determine what is best for the student group in front of them and how to respond with appropriate behaviors and models.

The design and implementation of the program can either enhance or inhibit the development of character strengths, with results varying across different contexts. Depending on the targeted and prioritized character strengths, different combinations of levers will prove more effective than others. For instance, high levels of authentic adventure and low levels of service might increase opportunities to develop performance character and demphasize opportunities to develop moral or civic character. By acknowledging the constraints of the operational context, instructors can respond by adjusting certain behaviors or models, such as reflection, roles, and chores, or comfort zone models. These adjustments can intentionally emphasize character strengths that are less inherent in the program’s design, accounting for the less malleable aspects of a school’s operating context.

We also observed less effective lever use, which generally meant that observed design and implementation decisions were seemingly misaligned with other levers being used. This was often the case in newer Schools that had borrowed or co-developed certain levers from older Schools, but differences in Context factors did not support the adopted levers. For example, common observations of this misalignment included a lesser design of authentic adventure (e.g., primarily center-based programs) combined with educational models better suited to journey-based programs (e.g., training-main-final progression). While specific design or implementation decisions can be justified by outcomes desired or the student group, misalignment or a lack of coherence between levers can leave the audience confused without a clear course arc or theme and can lead to less effective character development.

### Implications for character education

While there has been a great deal of recent attention on how character education operates in formal schooling [6,19,21], out-of-school experiences generally and outdoor education specifically have largely been omitted from this work on the science of character, despite a rich legacy of character development work [22,23]. While many of the drivers of successful character education programs can be applied to OB or outdoor education more generally, such programs also have distinct features that make them differently effective in adding to the landscape of character development work. First, programs such as OB explicitly target character development as the primary outcome of their courses. Character is prioritized and is not forced to compete for time, attention, and teacher competence with traditional academic subjects such as math or science. Second, the residential nature of these programs elevates both relationship development and instructional autonomy. Instructional staff often live and eat in a shared community with their students and are able to make adjustments to the course to accommodate student needs. If extra time is needed to reflect on an especially fruitful activity, instructors can often adjust the course schedule without the tight time constraints of many traditional schools. Third, out-of-school time activities are more effectively able to incorporate meaningful service and authentic adventure into their programming, both of which we identified as powerful levers for character development in this study. Thus, this study further illustrates the continued value of outdoor education to complement traditional schooling [24] and proffers some specific strengths for character education less accessible in more traditional educational settings.

### Implications for global character work

Similar to other global organizations that operate across cultures, OBI must navigate a balance between standardization and oversight that appreciates local contributions, connections, and adaptations. One reasonable parallel that has been examined in formal schooling is the International Baccalaureate (IB) program. IB is an emblematic case of educational globalization and offers insights into global oversight and local delivery of education, where structure and practices operate to transcend regional values and educational systems [25]. Like OB, IB has flourished in some countries and educational systems and struggled with integration in others [26].

In discussing the IB model, Resnik [27] proffers two key concepts necessary for the successful expansion and scaling of a global education network: insertion and adaptation. Insertion strategies refer to the integration of programs with regional and educational policies and local values. In our study, the OB pedagogy must hold value and benefits in terms of quality control, reputation, and contribution to youth education, lest efforts to insert it into existing systems would not be justified. Adaptation strategies refer to changes made to a program that are necessary for success within an individual School’s operational context. In OB’s case, the oversight provided by the global network balances the needs for adaptation without losing the essence that bolsters OB’s reputation for quality and efficacy in character development. Flexibility in requirements and attention to local needs are what have allowed OB to expand. Valued and beneficial guidance inserted by the network should be coupled with flexible and adaptable application in diverse operational contexts.

Understanding and valuing local expertise and needs is critical to global reach and expansion. Expertise “lives” at individual Schools, and this expertise shapes how the global entity (OBI) operates and influences education, resources, and oversight. There is a dynamic interplay between the global oversight and local expertise (contextual needs), either of which can facilitate or thwart successful global expansion.

A key finding from our study is that while insertion and adaptation are essential strategies for building a global education network, they must operate within a broader commitment to coherence. When most effective, these strategies are intentionally designed to align pedagogical processes and desired character outcomes so that local adaptations do not undermine the integrity of the educational approach. Although coherence in teaching and instruction is not a new concept [28], it can be difficult to maintain when organizations prioritize flexibility during local implementation. Without a unifying structure, what emerges is often a loose collection of philosophies and programmatic elements that may appear reasonable on their own but fail to function together in a coherent whole.

Maintaining programmatic coherence requires grounding educational efforts in a clear philosophy and a robust theory of change, which reduces the fragmentation that often occurs when programs are assembled from disparate “OB activities” placed into unfamiliar contexts [29]. Even when developed by passionate and dedicated educators, such initiatives can falter if they lack an overarching, coherent plan.

Research consistently underscores the value of coherence, demonstrating its positive influence on student outcomes [30,31], on learning transfer [32], and on student motivation [33]. These findings highlight that coherence is not merely an organizational preference but a critical component of effective educational design and implementation.

### Implications for contemporary Outward Bound pedagogy and practice

Finally, the proposed theoretical framework can serve as a platform to invite pedagogical coherence both within the OB Schools and throughout the OBI network. Integration with the existing OBI PPP approach will aid adoption and dialogue. Some obvious links exist, such as authentic adventure remaining at the intersection of Process and Places, and educational models existing within the Process. We propose that the entire PPP approach operates within a context and educational philosophy, which informs the design and implementation of all subsequent levers. As highlighted in our results, understanding the School’s context and philosophy and informing course design through this understanding is important for coherent course delivery. We see instructor behaviors occupying People and the intersections of People and Places, and the intersection of People and Process. Certain instructor behaviors, such as student-instructor relationships, best exemplify People and Process. In contrast, other behaviors, such as instructor autonomy or time management, could exist at the intersection of People and Places. Instructors must understand how their pedagogy is aligned with other levers at their School. Even strong pedagogical practices, when misaligned with other levers in use, may lose their effectiveness. Finally, we believe service exists at the intersection of Places and Process. Key to the design phase of course, which incorporates Service as part of the Process, and ideally integrated into local communities, which situates Service in Place.

By reflecting on the implications of this study, OBI and character development efforts across the network can more effectively balance contextual relevance with meaningful oversight and quality assurance. When programs intentionally incorporate context-specific knowledge alongside research- and practice-based forms of evidence [34], they create the conditions for high-quality, culturally responsive character education to flourish within outdoor learning environments.

## Limitations

This research is not without limitations. While methodical, case-study selection was limited by Schools’ voluntary participation and capacity to host the research team. This could privilege more well-resourced Schools across the network, and eliminated some Schools, such as those located on the continent of Africa, from inclusion. This structural constraint of our approach likely limits the transferability of our findings to Schools with at least moderate organizational capacity.

Given the multi-year, partnership-based nature of the research and the need to maintain access across a global network of sites, this study adopts a collaborative, strengths-based approach to examining practice across OB Schools. Accordingly, the study focuses on how key elements were expressed across cases, rather than systematically evaluating or comparing the relative effectiveness of individual Schools. While positive or neutral examples are provided for specific School locations, deviant examples that could be considered detrimental to Schools are only reported in aggregate. This approach supports the development of a cross-case framework while maintaining the relational and ethical commitments necessary for conducting in-depth, field-based research in applied settings.

In addition, single-course-type observations limited the richness of observational data from each School. Observing multiple course types (e.g., different populations served, length, ages, instructors) at each School would increase observer understanding of each School’s design and implementation range.

Future research using complementary designs may more directly examine variation in outcomes across contexts and test the relationships proposed in this framework. Future comparisons of more polar School types would broaden the examination of School practices.

Finally, the challenge of multi-lingual course delivery, despite fairly consistent availability of interpreters, limited researchers’ ability to understand elements of course implementation in action. Greater linguistic diversity in the research team, all native English speakers, could combat this limitation for future research.

## Conclusion

Although all OB Schools engage in character development, they do so in ways that reflect their distinct operational contexts. The propositions developed in this study help explain how Schools effectively customize their programming to address the character needs of the contexts in which they operate. While theory building through a cross-case study approach offers a valuable foundation, continued testing of these propositions is essential. Each proposition, individually and collectively, presents meaningful opportunities for future research both within OB and across the broader field of outdoor education.

What emerges clearly from this work is that OB provides powerful and varied pathways for fostering character in young people around the world. Despite differences in how character is conceptualized, which strengths are emphasized, and the methods used to cultivate them, this study illustrates that OB has multiple levers it can adjust to support character development. By deepening our understanding of how to tune these levers to enhance coherence and maximize impact, this research contributes to improving the quality and effectiveness of character education over time.
