# Supplementary material for: Developing an evidence-based framework of contemporary character development at Outward Bound Schools
Source: PLoS One. 2026 Jun 12;21(6):e0351185. doi: 10.1371/journal.pone.0351185 (PMC13262807; doi:10.1371/journal.pone.0351185)
Supplement: Attachment — Submitted filename: Cover Resubmission Letter to the Reviewers.docx [file pone.0351185.s001.docx]

Dear Dr. Chan,

Thank you for the thoughtful review of our paper, “Developing an evidence-based framework of contemporary character development at Outward Bound Schools.” We appreciate the opportunity to improve our submission based on feedback from you and the reviewer. We have retained the numbering system from your decision letter below so that you can more easily track our responses. All line numbers referenced below are related to the Manuscript with Tracked Changes so that edits can be easily viewed.

**Journal Requirements:**

1,2. We have addressed the formatting and financial disclosure concerns. The statement you have offered is correct: "The funders had no role in study design, data collection and analysis, decision to publish, or preparation of the manuscript." Thank you for changing it in the online submission form.

3. We have added our ethics statement in the Methods section, including the full name of the IRB that reviewed our study (Penn State’s Office for Research Protections; see first paragraph of Methods). We obtained written informed consent from the guardians of minors, followed by verbal assent from the minors and verbal informed consent from adults.

4. Much of our data has been placed on the Hive data repository at the University of Utah (<https://doi.org/10.7278/S5d-kf6w-aqha>). We have shared all that we are ethically allowed to share publicly and revised our Data Availability Statement in the submission.

5: There were no additional citations suggested by the reviewers, and we have cross-checked the current reference list as well as corrected it to reflect additions made through revisions.

**Response to Editor and Reviewer Comments:**

The review comments highlight five main concerns (First-Fifth), which we have numbered 1-5 below.

1. Clarification and Expansion of Data Analysis Procedures

We appreciate the reviewer’s request for greater transparency in the data analysis process. In response, we have substantially expanded the Methods section (lines 210-245) to provide a clearer account of how data were coded, analyzed, and consolidated into the proposed framework.

Specifically, we now:

- Share additional information on how field notes and interviews were developed into case study files, which were used for thematic analysis.
- Clarify the process of reflective thematic analysis and add citations to Braun and Clarke’s work (2006; 2019) that informed the process of coding and theme construction.
- Explain the process of axial coding, including how relationships among codes were identified and organized into higher-order propositions relating constructs.
- Provide additional detail on how the research team discussed the coding process, including the consensus approach that was utilized to resolve discrepancies in developing propositions.

We have also added illustrative examples linking data excerpts to emerging themes to strengthen transparency and demonstrate the connection between empirical observations and the resulting propositions.

2. Strengthening the Link Between Data and Propositions (Cross-Case Patterns)

First, we have softened the causal language (“enhancing or more effective); see also 3 below. Further, we have clarified that our approach was to focus on recurring patterns in design and implementation that we observed alongside evidence-based character education practices rather than to link specific actions to character outcomes.

Regarding deviant or counterexamples, we have added additional counterexamples (see lines 410-417, 455-464, 522-529, and 571-586 as examples) at the end of each proposition sections. As this was a multi-year research-practice collaboration, we have committed to individual Schools to publicly highlight only neutral or positive areas, reporting negative or deviant examples anonymously or in aggregate. However, decontextualizing them does erode some of our evidence. We have noted this as a limitation in the limitations section (lines 923-931).

3. Revision of Causal Language and Framing as Theory-Building

We thank the reviewer for this important observation and have revised the manuscript to more clearly align our claims with the theory-building nature of the study. We did not intend to imply that this paper provided evidence of character development or that we had empirically linked the levers identified in the paper to operationalized measures of character. Our intent was always to propose a framework based on observed and reported co-occurrences (association) between observed design and implementation factors (our levers) and opportunities for character growth, as aligned with OB’s history and models and related literature. Therefore, we have softened the causal language throughout the paper and clarified our intent as theory building rather than evidence of effectiveness, as suggested by the reviewer. See rewording in each proposition as well as tracked changes throughout the paper.

In particular, we have:

- Reframed the propositions to emphasize patterns and associations rather than causal or effectiveness claims (e.g., replacing language such as “enhances” or “increases” with “is associated with,” “appears to support,” or “was observed alongside”).
- Removed or revised evaluative language (e.g., “more effective” / “less effective”) and replaced it with descriptions of variation in how program features were expressed across cases.
- Revised sections of the paper to more explicitly state that the study identifies recurring patterns and proposes relationships, rather than testing causal effects (e.g., see lines 316-318 and 373).

These revisions ensure that the conclusions are appropriately calibrated to the data and consistent with an Eisenhardt theory-building approach.

4. Addressing Sampling and Selection Bias

We agree with the reviewer and have specifically noted this as a limitation (see lines 918-922).

5. Addition of Reflexivity and Positionality Statement

We agree with the reviewer and have specifically added a reflexivity and positionality statement in the Methods section (see lines 247-270).

Finally, we have made efforts to clarify our writing and intent throughout. We have worked to reduce evaluative language (e.g., as noted by the reviewer, “more effective, less effective”). This was necessary as we clarified the intent to identify cross-case patterns rather than to provide evidence of effectiveness, resulting in propositions, which combine observations and interpretations into testable relationships. We have made these revisions to more clearly orient the reader to our study purpose. In addition to the substantive edits above, we have made smaller editorial adjustments to enhance clarity and, where appropriate, throughout the paper.

Sincerely,

Jim Sibthorp (jim.sibthorp@health.utah.edu), Nick Rushford, Pete Allison, Theresa Melton, and Sarah Wiley
