# Supplementary material for: Developing an evidence-based framework of contemporary character development at Outward Bound Schools
Source: PLoS One. 2026 Jun 12;21(6):e0351185. doi: 10.1371/journal.pone.0351185 (PMC13262807; doi:10.1371/journal.pone.0351185)
Supplement: Peer review history

# Peer review: Decision Letter 0

**DOI:** 10.1371/journal.pone.0351185.r001

**Authors:** Rachel Suet Kay Chan

19 Mar 2026

PONE-D-26-04562

Developing an evidence-based framework of contemporary character development at Outward Bound Schools

PLOS One

Dear Dr. Sibthorp,

Thank you for submitting your manuscript to PLOS ONE. After careful consideration, we feel that it has merit but does not fully meet PLOS ONE’s publication criteria as it currently stands. Therefore, we invite you to submit a revised version of the manuscript that addresses the points raised during the review process.

==============================

Dear Author

The reviewer/s have recommended a Minor Revision.

Please refer to their comments below.

Thank you.

==============================

Please submit your revised manuscript by May 03 2026 11:59PM. If you will need more time than this to complete your revisions, please reply to this message or contact the journal office at plosone@plos.org. When you're ready to submit your revision, log on to https://www.editorialmanager.com/pone/ and select the 'Submissions Needing Revision' folder to locate your manuscript file.

Please include the following items when submitting your revised manuscript:

- A letter that responds to each point raised by the academic editor and reviewer(s). You should upload this letter as a separate file labeled 'Response to Reviewers'.
- A marked-up copy of your manuscript that highlights changes made to the original version. You should upload this as a separate file labeled 'Revised Manuscript with Track Changes'.
- An unmarked version of your revised paper without tracked changes. You should upload this as a separate file labeled 'Manuscript'.

-->If you would like to make changes to your financial disclosure, please include your updated statement in your cover letter. Guidelines for resubmitting your figure files are available below the reviewer comments at the end of this letter.

If applicable, we recommend that you deposit your laboratory protocols in protocols.io to enhance the reproducibility of your results. Protocols.io assigns your protocol its own identifier (DOI) so that it can be cited independently in the future. For instructions see: https://journals.plos.org/plosone/s/submission-guidelines#loc-laboratory-protocols. Additionally, PLOS ONE offers an option for publishing peer-reviewed Lab Protocol articles, which describe protocols hosted on protocols.io. Read more information on sharing protocols at https://plos.org/protocols?utm_medium=editorial-email&utm_source=authorletters&utm_campaign=protocols.

We look forward to receiving your revised manuscript.

Kind regards,

Rachel Suet Kay Chan, Ph.D.

Academic Editor

PLOS One

Journal Requirements:

When submitting your revision, we need you to address these additional requirements.

1. Please ensure that your manuscript meets PLOS ONE's style requirements, including those for file naming. The PLOS ONE style templates can be found at

https://journals.plos.org/plosone/s/file?id=wjVg/PLOSOne_formatting_sample_main_body.pdf and

https://journals.plos.org/plosone/s/file?id=ba62/PLOSOne_formatting_sample_title_authors_affiliations.pdf

2. Thank you for stating the following financial disclosure:

“This project/ publication was made possible through the support of Grant 62329 from the John Templeton Foundation. The opinions expressed in this publication are those of the authors and do not necessarily reflect the views of the John Templeton Foundation.”

Please state what role the funders took in the study.  If the funders had no role, please state: "The funders had no role in study design, data collection and analysis, decision to publish, or preparation of the manuscript."

If this statement is not correct you must amend it as needed.

Please include this amended Role of Funder statement in your cover letter; we will change the online submission form on your behalf.

3. Please include your full ethics statement in the ‘Methods’ section of your manuscript file. In your statement, please include the full name of the IRB or ethics committee who approved or waived your study, as well as whether or not you obtained informed written or verbal consent. If consent was waived for your study, please include this information in your statement as well.

4. We note that you have indicated that there are restrictions to data sharing for this study. PLOS only allows data to be available upon request if there are legal or ethical restrictions on sharing data publicly. For more information on unacceptable data access restrictions, please see http://journals.plos.org/plosone/s/data-availability#loc-unacceptable-data-access-restrictions.

Before we proceed with your manuscript, please address the following prompts:

a) If there are ethical or legal restrictions on sharing a de-identified data set, please explain them in detail (e.g., data contain potentially identifying or sensitive patient information, data are owned by a third-party organization, etc.) and who has imposed them (e.g., a Research Ethics Committee or Institutional Review Board, etc.). Please also provide contact information for a data access committee, ethics committee, or other institutional body to which data requests may be sent.

b) If there are no restrictions, please upload the minimal anonymized data set necessary to replicate your study findings to a stable, public repository and provide us with the relevant URLs, DOIs, or accession numbers. For a list of recommended repositories, please see

https://journals.plos.org/plosone/s/recommended-repositories. You also have the option of uploading the data as Supporting Information files, but we would recommend depositing data directly to a data repository if possible.

We will update your Data Availability statement on your behalf to reflect the information you provide

5. If the reviewer comments include a recommendation to cite specific previously published works, please review and evaluate these publications to determine whether they are relevant and should be cited. There is no requirement to cite these works unless the editor has indicated otherwise.

Please review your reference list to ensure that it is complete and correct. If you have cited papers that have been retracted, please include the rationale for doing so in the manuscript text, or remove these references and replace them with relevant current references. Any changes to the reference list should be mentioned in the rebuttal letter that accompanies your revised manuscript. If you need to cite a retracted article, indicate the article’s retracted status in the References list and also include a citation and full reference for the retraction notice.

Additional Editor Comments:

Dear Author

The reviewer/s have recommended a Minor Revision.

Please refer to their comments below.

Thank you

[Note: HTML markup is below. Please do not edit.]

Reviewers' comments:

Reviewer's Responses to Questions

**Comments to the Author**

1. Is the manuscript technically sound, and do the data support the conclusions?

The manuscript must describe a technically sound piece of scientific research with data that supports the conclusions. Experiments must have been conducted rigorously, with appropriate controls, replication, and sample sizes. The conclusions must be drawn appropriately based on the data presented.

Reviewer #1: Partly

**********

2. Has the statistical analysis been performed appropriately and rigorously?

Reviewer #1: N/A

**********

3. Have the authors made all data underlying the findings in their manuscript fully available?

The PLOS Data policy requires authors to make all data underlying the findings described in their manuscript fully available without restriction, with rare exception (please refer to the Data Availability Statement in the manuscript PDF file). The data should be provided as part of the manuscript or its supporting information, or deposited to a public repository. For example, in addition to summary statistics, the data points behind means, medians and variance measures should be available. If there are restrictions on publicly sharing data—e.g. participant privacy or use of data from a third party—those must be specified.

Reviewer #1: Yes

**********

4. Is the manuscript presented in an intelligible fashion and written in standard English?

PLOS ONE does not copyedit accepted manuscripts, so the language in submitted articles must be clear, correct, and unambiguous. Any typographical or grammatical errors should be corrected at revision, so please note any specific errors here.

Reviewer #1: Yes

**********

5. Review Comments to the Author

Please use the space provided to explain your answers to the questions above. You may also include additional comments for the author, including concerns about dual publication, research ethics, or publication ethics. (Please upload your review as an attachment if it exceeds 20,000 characters)

Reviewer #1: The manuscript presents an interesting and potentially valuable qualitative, multi-case study aimed at developing a framework for character development across Outward Bound schools globally. The use of an Eisenhardt multiple-case, cross-case design is appropriate for theory-building purposes, and the inclusion of diverse international cases is a notable strength. The study also benefits from multiple data sources (observations, interviews, surveys, and photovoice) and from efforts toward triangulation and member checking. However, several aspects of the methodological rigor and the linkage between data and conclusions require clarification and strengthening to meet the standards of technical soundness expected by PLOS ONE.

First, the description of the data analysis process is insufficiently detailed. While the manuscript mentions independent and axial coding, it does not provide sufficient transparency on how codes were developed, refined, and translated into themes, and ultimately into the five proposed “levers.” The authors should provide a clearer account of the analytical procedure, including whether a coding framework or codebook was developed, how disagreements between researchers were resolved, and how decisions were made to move from codes to higher-order constructs. Including examples of coded data or excerpts from field notes/interviews linked to specific themes would substantially improve transparency and credibility.

Second, the manuscript would benefit from a stronger, more explicit connection between the empirical data and the resulting propositions. Currently, many claims (e.g., that certain levers “enhance” character development or that some approaches are “more effective”) are presented without sufficient systematic evidence. The findings rely heavily on illustrative anecdotes rather than demonstrating patterns across cases. The authors are encouraged to provide more structured cross-case comparisons (e.g., tables or matrices showing how each lever was observed across the 11 cases) and to indicate whether there were any counterexamples or deviant cases. This would help readers assess the robustness of the findings and reduce the risk of overgeneralization.

Third, the strength of the conclusions appears to exceed what the data can support. The propositions are framed in a way that suggests effectiveness (e.g., “enhances character development opportunities”). Yet, the study does not include direct measures of character development outcomes nor systematic comparisons of impact across cases. As such, the conclusions should be more cautiously framed as exploratory, hypothesis-generating, or theory-building rather than as evidence of effectiveness. Alternatively, the authors should more clearly explain the basis for inferring effectiveness.

Fourth, there are potential concerns related to sampling and selection bias that should be addressed more explicitly. Case selection was partly dependent on schools’ willingness and capacity to participate, which may bias the sample toward better-resourced or more engaged institutions. The implications of this for the generalizability or transferability of the findings should be discussed in greater depth.

Fifth, while the manuscript notes efforts to mitigate researcher bias (e.g., multiple researchers, expert interviews, member checking), it lacks a reflexivity statement describing the researchers’ positionality and how it may have influenced data collection and interpretation. Given the interpretive nature of the study, this would strengthen methodological rigor.

Finally, the manuscript would benefit from clearer distinctions among observation, interpretation, and inference throughout the results section. In several instances, evaluative language (e.g., “more effective,” “less effective”) is used without clearly specifying the evidentiary basis. Clarifying how such judgments were derived from the data would improve the study's internal validity.

In summary, the study addresses an important topic. It employs an appropriate qualitative design, but revisions are needed to enhance analytical transparency, strengthen the link between the data and the conclusions, and ensure that claims are adequately supported by the evidence presented.

**********

6. PLOS authors have the option to publish the peer review history of their article (what does this mean?). If published, this will include your full peer review and any attached files.

If you choose “no”, your identity will remain anonymous but your review may still be made public.

**Do you want your identity to be public for this peer review?** For information about this choice, including consent withdrawal, please see our Privacy Policy.

Reviewer #1: No

**********

[NOTE: If reviewer comments were submitted as an attachment file, they will be attached to this email and accessible via the submission site. Please log into your account, locate the manuscript record, and check for the action link "View Attachments". If this link does not appear, there are no attachment files.]

To ensure your figures meet our technical requirements, please review our figure guidelines: https://journals.plos.org/plosone/s/figures

You may also use PLOS’s free figure tool, NAAS, to help you prepare publication quality figures: https://journals.plos.org/plosone/s/figures#loc-tools-for-figure-preparation.

NAAS will assess whether your figures meet our technical requirements by comparing each figure against our figure specifications.

# Author comment: Author response to Decision Letter 1

**DOI:** 10.1371/journal.pone.0351185.r002

8 May 2026

Dear Dr. Chan,

Thank you for the thoughtful review of our paper, “Developing an evidence-based framework of contemporary character development at Outward Bound Schools.” We appreciate the opportunity to improve our submission based on feedback from you and the reviewer. We have retained the numbering system from your decision letter below so that you can more easily track our responses. All line numbers referenced below are related to the Manuscript with Tracked Changes so that edits can be easily viewed.

Journal Requirements:

1,2. We have addressed the formatting and financial disclosure concerns. The statement you have offered is correct: "The funders had no role in study design, data collection and analysis, decision to publish, or preparation of the manuscript." Thank you for changing it in the online submission form.

3. We have added our ethics statement in the Methods section, including the full name of the IRB that reviewed our study (Penn State’s Office for Research Protections; see first paragraph of Methods). We obtained written informed consent from the guardians of minors, followed by verbal assent from the minors and verbal informed consent from adults.

4. Much of our data has been placed on the Hive data repository at the University of Utah (https://doi.org/10.7278/S5d-kf6w-aqha). We have shared all that we are ethically allowed to share publicly and revised our Data Availability Statement in the submission.

5: There were no additional citations suggested by the reviewers, and we have cross-checked the current reference list as well as corrected it to reflect additions made through revisions.

Response to Editor and Reviewer Comments:

The review comments highlight five main concerns (First-Fifth), which we have numbered 1-5 below.

1. Clarification and Expansion of Data Analysis Procedures

We appreciate the reviewer’s request for greater transparency in the data analysis process. In response, we have substantially expanded the Methods section (lines 210-245) to provide a clearer account of how data were coded, analyzed, and consolidated into the proposed framework.

Specifically, we now:

• Share additional information on how field notes and interviews were developed into case study files, which were used for thematic analysis.

• Clarify the process of reflective thematic analysis and add citations to Braun and Clarke’s work (2006; 2019) that informed the process of coding and theme construction.

• Explain the process of axial coding, including how relationships among codes were identified and organized into higher-order propositions relating constructs.

• Provide additional detail on how the research team discussed the coding process, including the consensus approach that was utilized to resolve discrepancies in developing propositions.

We have also added illustrative examples linking data excerpts to emerging themes to strengthen transparency and demonstrate the connection between empirical observations and the resulting propositions.

2. Strengthening the Link Between Data and Propositions (Cross-Case Patterns)

First, we have softened the causal language (“enhancing or more effective); see also 3 below. Further, we have clarified that our approach was to focus on recurring patterns in design and implementation that we observed alongside evidence-based character education practices rather than to link specific actions to character outcomes.

Regarding deviant or counterexamples, we have added additional counterexamples (see lines 410-417, 455-464, 522-529, and 571-586 as examples) at the end of each proposition sections. As this was a multi-year research-practice collaboration, we have committed to individual Schools to publicly highlight only neutral or positive areas, reporting negative or deviant examples anonymously or in aggregate. However, decontextualizing them does erode some of our evidence. We have noted this as a limitation in the limitations section (lines 923-931).

3. Revision of Causal Language and Framing as Theory-Building

We thank the reviewer for this important observation and have revised the manuscript to more clearly align our claims with the theory-building nature of the study. We did not intend to imply that this paper provided evidence of character development or that we had empirically linked the levers identified in the paper to operationalized measures of character. Our intent was always to propose a framework based on observed and reported co-occurrences (association) between observed design and implementation factors (our levers) and opportunities for character growth, as aligned with OB’s history and models and related literature. Therefore, we have softened the causal language throughout the paper and clarified our intent as theory building rather than evidence of effectiveness, as suggested by the reviewer. See rewording in each proposition as well as tracked changes throughout the paper.

In particular, we have:

• Reframed the propositions to emphasize patterns and associations rather than causal or effectiveness claims (e.g., replacing language such as “enhances” or “increases” with “is associated with,” “appears to support,” or “was observed alongside”).

• Removed or revised evaluative language (e.g., “more effective” / “less effective”) and replaced it with descriptions of variation in how program features were expressed across cases.

• Revised sections of the paper to more explicitly state that the study identifies recurring patterns and proposes relationships, rather than testing causal effects (e.g., see lines 316-318 and 373).

These revisions ensure that the conclusions are appropriately calibrated to the data and consistent with an Eisenhardt theory-building approach.

4. Addressing Sampling and Selection Bias

We agree with the reviewer and have specifically noted this as a limitation (see lines 918-922).

5. Addition of Reflexivity and Positionality Statement

We agree with the reviewer and have specifically added a reflexivity and positionality statement in the Methods section (see lines 247-270).

Finally, we have made efforts to clarify our writing and intent throughout. We have worked to reduce evaluative language (e.g., as noted by the reviewer, “more effective, less effective”). This was necessary as we clarified the intent to identify cross-case patterns rather than to provide evidence of effectiveness, resulting in propositions, which combine observations and interpretations into testable relationships. We have made these revisions to more clearly orient the reader to our study purpose. In addition to the substantive edits above, we have made smaller editorial adjustments to enhance clarity and, where appropriate, throughout the paper.

Sincerely,

Jim Sibthorp (jim.sibthorp@health.utah.edu), Nick Rushford, Pete Allison, Theresa Melton, and Sarah Wiley

Attachment Submitted filename: Cover Resubmission Letter to the Reviewers.docx

# Editor report: Decision Letter 1

**DOI:** 10.1371/journal.pone.0351185.r003

**Authors:** Rachel Suet Kay Chan

24 May 2026

Developing an evidence-based framework of contemporary character development at Outward Bound Schools

PONE-D-26-04562R1

Dear Dr. Sibthorp,

We’re pleased to inform you that your manuscript has been judged scientifically suitable for publication and will be formally accepted for publication once it meets all outstanding technical requirements.

Within one week, you’ll receive an e-mail detailing the required amendments. When these have been addressed, you’ll receive a formal acceptance letter and your manuscript will be scheduled for publication.

An invoice will be generated when your article is formally accepted. Please note, if your institution has a publishing partnership with PLOS and your article meets the relevant criteria, all or part of your publication costs will be covered. Please make sure your user information is up-to-date by logging into Editorial Manager at Editorial Manager® and clicking the ‘Update My Information' link at the top of the page. For questions related to billing, please contact billing support.

If your institution or institutions have a press office, please notify them about your upcoming paper to help maximize its impact. If they’ll be preparing press materials, please inform our press team as soon as possible -- no later than 48 hours after receiving the formal acceptance. Your manuscript will remain under strict press embargo until 2 pm Eastern Time on the date of publication. For more information, please contact onepress@plos.org.

Kind regards,

Rachel Suet Kay Chan, Ph.D.

Academic Editor

PLOS One

Additional Editor Comments (optional):

Reviewers' comments:

# Editor report: Acceptance letter

**DOI:** 10.1371/journal.pone.0351185.r004

**Authors:** Rachel Suet Kay Chan

PONE-D-26-04562R1

PLOS One

Dear Dr. Sibthorp,

I'm pleased to inform you that your manuscript has been deemed suitable for publication in PLOS One. Congratulations! Your manuscript is now being handed over to our production team.

At this stage, our production department will prepare your paper for publication. This includes ensuring the following:

* All references, tables, and figures are properly cited

* All relevant supporting information is included in the manuscript submission,

* There are no issues that prevent the paper from being properly typeset

You will receive further instructions from the production team, including instructions on how to review your proof when it is ready. Please keep in mind that we are working through a large volume of accepted articles, so please give us a few days to review your paper and let you know the next and final steps.

Lastly, if your institution or institutions have a press office, please let them know about your upcoming paper now to help maximize its impact. If they'll be preparing press materials, please inform our press team within the next 48 hours. Your manuscript will remain under strict press embargo until 2 pm Eastern Time on the date of publication. For more information, please contact onepress@plos.org.

You will receive an invoice from PLOS for your publication fee after your manuscript has reached the completed accept phase. If you receive an email requesting payment before acceptance or for any other service, this may be a phishing scheme. Learn how to identify phishing emails and protect your accounts at https://explore.plos.org/phishing.

If we can help with anything else, please email us at customercare@plos.org.

Thank you for submitting your work to PLOS ONE and supporting open access.

Kind regards,

PLOS ONE Editorial Office Staff

on behalf of

Dr. Rachel Suet Kay Chan

Academic Editor

PLOS One
